# EEG-Based Mental Workload Neurometric to Evaluate the Impact of Different Traffic and Road Conditions in Real Driving Settings

**DOI:** 10.3389/fnhum.2018.00509

**Published:** 2018-12-18

**Authors:** Gianluca Di Flumeri, Gianluca Borghini, Pietro Aricò, Nicolina Sciaraffa, Paola Lanzi, Simone Pozzi, Valeria Vignali, Claudio Lantieri, Arianna Bichicchi, Andrea Simone, Fabio Babiloni

**Affiliations:** ^1^BrainSigns srl, Rome, Italy; ^2^IRCCS Fondazione Santa Lucia, Neuroelectrical Imaging and BCI Lab, Rome, Italy; ^3^Department of Molecular Medicine, Sapienza University of Rome, Rome, Italy; ^4^Department of Anatomical, Histological, Forensic and Orthopedic Sciences, Sapienza University of Rome, Rome, Italy; ^5^Deep Blue srl, Rome, Italy; ^6^Department of Civil, Chemical, Environmental and Materials Engineering (DICAM), School of Engineering and Architecture, University of Bologna, Bologna, Italy; ^7^Department of Computer Science, Hangzhou Dianzi University, Hangzhou, China

**Keywords:** electroencephalography, mental workload, human factor, machine-learning, asSWLDA, neuroergonomics, car driving, road safety

## Abstract

Car driving is considered a very complex activity, consisting of different concomitant tasks and subtasks, thus it is crucial to understand the impact of different factors, such as road complexity, traffic, dashboard devices, and external events on the driver’s behavior and performance. For this reason, in particular situations the cognitive demand experienced by the driver could be very high, inducing an excessive experienced mental workload and consequently an increasing of error commission probability. In this regard, it has been demonstrated that human error is the main cause of the 57% of road accidents and a contributing factor in most of them. In this study, 20 young subjects have been involved in a real driving experiment, performed under different traffic conditions (rush hour and not) and along different road types (main and secondary streets). Moreover, during the driving tasks different specific events, in particular a pedestrian crossing the road and a car entering the traffic flow just ahead of the experimental subject, have been acted. A Workload Index based on the Electroencephalographic (EEG), i.e., brain activity, of the drivers has been employed to investigate the impact of the different factors on the driver’s workload. Eye-Tracking (ET) technology and subjective measures have also been employed in order to have a comprehensive overview of the driver’s perceived workload and to investigate the different insights obtainable from the employed methodologies. The employment of such EEG-based Workload index confirmed the significant impact of both traffic and road types on the drivers’ behavior (increasing their workload), with the advantage of being under real settings. Also, it allowed to highlight the increased workload related to external events while driving, in particular with a significant effect during those situations when the traffic was low. Finally, the comparison between methodologies revealed the higher sensitivity of neurophysiological measures with respect to ET and subjective ones. In conclusion, such an EEG-based Workload index would allow to assess objectively the mental workload experienced by the driver, standing out as a powerful tool for research aimed to investigate drivers’ behavior and providing additional and complementary insights with respect to traditional methodologies employed within road safety research.

## Introduction

According to the reports of World Health Organization (WHO) (World Health Organization, 2015), every year traffic accidents cause the death of 1.3 million people around the world, and moreover about 50 million people suffer from a disability caused by accidents related to cars. By 2020, it is estimated that traffic accidents will be the fifth leading cause of death in the world, reaching 2.4 million deaths per year (World Health Organization, 2013). Among the principal causes of the car accidents and related mortality there is the human factor (Hansen, 2007; Subramanian, 2012). In particular, it has been demonstrated that human error is the main cause of the 57% of road accidents and a contributing factor in over 90% of them (Treat et al., 1979). Driver’s common errors are largely correlated to overload, distractions, tiredness, or the simultaneous realization of other activities during driving (Allnutt, 1987; Horowitz and Dingus, 1992; Summala and Mikkola, 1994; Petridou and Moustaki, 2000). In fact, the human performance decrease, and consequently the errors commission, are directly attributable to aberrant mental states, in particular the mental workload while degrading in overload, which is considered one of the most important human factor constructs in influencing performance (Reason, 2000; Parasuraman et al., 2008; Paxion et al., 2014). The model theorized by De Waard (1996), widely used in automotive psychological research, establishes the relation between task demands and performance depending on the driver workload. This model describes the driving activity with a hierarchy of tasks on three levels, the *strategical*, the *tactical* and the *operational*, each of them divided into different subtasks, describing the driving as a very complex and often high-demanding activity. Therefore, the cognitive resources required in very complex situations can exceed the available resources, leading to an increase of workload and to performance impairments (Robert, 1997; Paxion et al., 2014).

The aforesaid statistics and findings justify the increasing attention received by the Human Factor within the road safety research during the last decades. As well as in other human-centered domains such as aviation and industry (Vicente, 2013; Toppi et al., 2016; Vecchiato et al., 2016; Borghini et al., 2017a), psychological disciplines have been taken on a considerable scientific importance receiving more and more attention. They have become a fundamental instrument for understanding and interpreting the behavior of the driver (Bucchi et al., 2012), trying to provide cognitive models in order to predict and avoid unsafe actions as well as to understand the relationship between such unsafe behaviors and different factors related to traffic, road complexity, car equipment and external events. The most frequently adopted techniques in this research field are those based on questionnaires and interviews after large-scale experiments in naturalistic (i.e., real driving) and simulated (i.e., by using simulator) settings. They make it possible to acquire useful information for personality tests and profiles, they help to highlight and correct behavioral difficulties and, therefore, they shape the driver to have a safe relationship with driving in different conditions, and in particular in emergency situations, as well as to improve road and car design and adapt safety education with respect to the driver background (Cestac et al., 2014; Kaplan et al., 2015).

In order to increase the strength of such psychological research applied to road safety, this discipline could now benefit from recent advancements and outcomes coming from Neuroscience and Neuroergonomics. The field of the Neuroergonomics aims to study the relationship between the human behavior and the brain at work (Parasuraman and Rizzo, 2008). It provides a multidisciplinary translational approach that merges elements of neuroscience, cognitive psychology, human factors and ergonomics to study brain structure and function in everyday environments. Applied to the driving safety domain, a Neuroergonomic approach should allow to investigate the relationship between human mental behavior, performance and road safety, taking advantage from neurophysiological measures and providing a deeper understanding of human cognition and its role in decision making and possible error commission at the wheel (Lees et al., 2010). In fact, it is widely accepted in scientific literature the limit of using subjective measures alone, such as questionnaires and interview, because of their intrinsic subjective nature and the impossibility to catch the “unconscious” phenomena behind human behaviors (Gopher and Braune, 1984; Dienes, 2004; Wall et al., 2004; Aricò et al., 2017b). In this context, technological advancements enable the use of neurophysiological measures, for example the measure of brain activity, heart activity, eye movements, to obtain objective measures of specific mental states with low invasiveness (Aricò et al., 2017c). Among the several neuroimaging techniques, such as functional Magnetic Resonance and Magnetoencephalography, Electroencephalographic technique (EEG) has been demonstrated to be one of the best techniques to infer, even in real time, objective assessment of mental states and in particular the mental workload experienced by the user, since other than being a direct measure of brain activations, it is characterized by high temporal resolution, limited cost and invasiveness (Prinzel et al., 2000; Aricò et al., 2016b). EEG-based measures of drivers’ mental states have been already investigated during the recent decades in order to determine brain cues of incoming risky psychophysical states, e.g., fatigue, drowsiness, inattention, overload (Lin et al., 2005; Michail et al., 2008; Brookhuis and de Waard, 2010; Borghini et al., 2012, 2014; Maglione et al., 2014; Wang et al., 2015; Zhang et al., 2015; Kong et al., 2015, 2017), and to develop futuristic Human-Machine interaction solutions and automation (Kohlmorgen et al., 2007; Lin et al., 2009; Göhring et al., 2013; Aricò et al., 2015). Nevertheless, two important gaps are still present in this domain:

(1) the majority of neurophysiological studies about drivers’ behaviors have been conducted in simulated environments or in poor realistic settings, but it has been proven that same experimental tasks are perceived differently, in terms of mental workload, if performed in a simulator or in real environment (de Winter et al., 2014); also, not only the task perception but the driver behavior itself related to a specific condition could change if the same condition is reproduced in simulators or in a real scenario (Philip et al., 2005);
(2) in scientific literature there is still the lack of a synthetic EEG-based workload index to adopt in a systematic way within the road safety research, in order to integrate results coming from traditional techniques, such as subjective measures and car parameters analysis, with additional insights arising directly from drivers’ brain (Paxion et al., 2014). Several studies about EEG correlates of driver’s mental workload have been carried on, however experimental examples in real settings of a multimodal approach integrating neurophysiological with traditional measures are still lacking (Xing et al., 2018).

In this study, it has been investigated the possibility to adopt the approach recently developed and patented by the authors of this work (Aricò et al., 2016b, 2017a), to evaluate the mental workload experienced by car drivers by means of their EEG activity. More specifically, such an approach is based on a machine-learning method able to assess, even online and in high-realistic environments, the user’s mental workload through a synthetic index. The authors successfully employed and validated such approach in different aviation-related applications, such as adaptive automation (Aricò et al., 2016a), personnel training (Borghini et al., 2017c), personnel expertise evaluation (Borghini et al., 2017b), moreover highlighting the higher sensitivity of such measures compared with subjective ones (Di Flumeri et al., 2015; Aricò et al., 2016b). Furthermore, the feasibility of obtaining EEG-based measures of driver’s workload has already been validated through a pilot study of the present work conducted with eight subjects while performing a simplified version of the real driving task employed within the present work (Di Flumeri et al., 2018).

For the present work, 20 young subjects have been involved in a real driving task along urban roads, performed under different traffic conditions (rush hour and not) and going through different road types (main and secondary streets). Also, during the driving tasks specific events, in particular a pedestrian crossing the road and a car entering the traffic flow just ahead of the experimental subject, have been acted. During the experiments the drivers’ brain activity, through EEG technique, and eye movements, through Eye-Tracking (ET) devices, have been collected. In addition, subjective measures, car parameters (e.g., position, speed, etc.) and videos around the car have been gathered. Thanks to this multimodal approach, the present study aimed at:

• Validating the machine-learning approach developed by the authors also in automotive domain, through an experiment in high-realistic settings, i.e., real driving;
• Employing the EEG-based Workload index obtained from the hence validated approach to evaluate the impact of different factors, specifically the road complexity, the traffic intensity (depending on the hour of the day), and two specific events (a pedestrian crossing the road and a car entering in the traffic flow), on the drivers’ mental workload;
• Comparing the neurophysiological measures with eye movements and subjective ones, in order to provide evidence of the complementarity of the obtained insights.

In conclusion, the present work will explore the potential of integrating these new methodologies, i.e., neurophysiological measures, with traditional approaches in order to enhance and extent research on drivers’ behaviors and road safety.

## Materials and Methods

### The Experimental Protocol

Twenty male students (24.9 ± 1.8 years old, licensed from 5.9 ± 1 years, with a mean annual mileage of 10350 km/year) from the University of Bologna (Italy) have been recruited and involved on a voluntary basis in this study. They were selected in order to have a homogeneous experimental group in terms of age, sex, and driving expertise. The experiment was conducted following the principles outlined in the Declaration of Helsinki of 1975, as revised in 2000. Informed consent and authorization to use the video graphical material were obtained from each subject on paper, after the explanation of the study.

**FIGURE 1:**
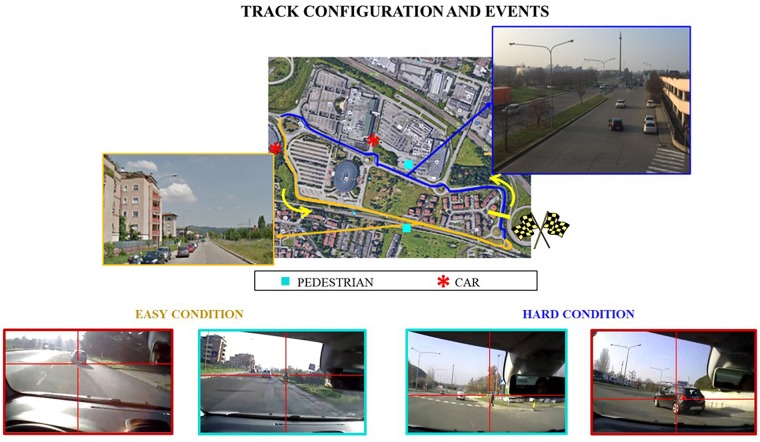
The experimental circuit about 2500 m long along Bologna roads. The blue line indicates the circuit segment labeled as “Hard” in terms of road complexity, while the yellow one the “Easy” segment. The cyan squares and the red asterisks represent the points were the events, respectively the pedestrian and the car, have been acted along the 3rd lap of both the task repetitions.

Two equal cars have been used for the experiments, i.e., Fiat 500L 1.3 Mjt, with diesel engine and manual transmission. The subjects had to drive the car along a route going through urban roads at the periphery of Bologna (Italy). In particular, the route consisted in three laps of a “circuit” about 2500 m long to be covered with the daylight (Figure 1).

The circuit was designed with the aim to include two segments of interest, both about 1000 m long but different in term of road complexity and so supposed different also in terms of cognitive demand, thus named hereafter “*Easy*” and “*Hard*”: (i) Easy was a secondary road, mainly straight, with an intersection halfway with the right-of-way, one lane and low traffic capacity, serving a residential area; (ii) Hard was a main road, mainly straight, with two roundabouts halfway, three lanes and high traffic capacity, serving a commercial area. This factor will be hereafter named “*ROAD.*” This assumption has been made on the basis of several evidences coming from scientific literature about road safety and behavior (Harms, 1991; Verwey, 2000; Paxion et al., 2014).

Furthermore, each subject had to repeat the task two times within the same day, one time during rush and one during normal hour: this factor will be hereafter named “*HOUR*,” while the two conditions “*Rush*” and “*Normal.*” The rush hours of that specific area have been determined according to the General Plan of Urban Traffic of Bologna (PGTU, please see Table 1): the two “Rush hour” time-windows were from 12:30 to 13:30 (lunchtime) and from 16:30 to 17:30 (work closing time), with the experiments performed from 9.30 to 17.30, in order to ensure a homogeneous daylight condition.

**Table 1:** Data extracted from the General Plan of Urban Traffic of Bologna (Italy) referred to the traffic flow intensities in the experimental area during the day.

Transits	Total	RUSH hour		NORMAL
	14 h (6 ÷ 20)	Morning (12:30–13:30)	Afternoon (16:30–17:30)	12 h
Total	19385	2024	2066	15295
Frequency (Transits/hour)	–	2024	2066	1274,6

*These data have been used to design two experimental conditions different in terms of traffic: the RUSH hours are characterized by traffic higher than during NORMAL hours.*

Finally, during the last lap (i.e., the 3rd one) of each task repetition (i.e., Rush and Normal hour) two different events have been simulated, by involving actors, twice (i.e., along the Hard and the Easy circuit segment) along the route: a pedestrian crossing the road, and a car entering the traffic flow just ahead of the experimental subject, hereafter labeled respectively “*Pedestrian*” and “*Car.*” The event types have been selected as the most probable events coherently with the urban context, as well as the safest to act, i.e., without introducing any risk for the actors, for the experimental subjects and for the traffic in general.

The Figure 1 shows the experimental circuit along Bologna roads, highlighting the “ROAD complexity” distribution as well as the occurred events.

To summarize, each subject, after a proper experimental briefing, performed a driving task of three laps along a circuit through urban roads two times, during Rush and Normal hours. The order of Rush and Normal conditions has been randomized among the subjects, in order to avoid any order effect (Kirk, 2015). Each lap consisted in a Hard and an Easy segment, where hard and easy are referred to the road complexity and thus task difficulty. Also, despite the initial briefing, the first lap of both the tasks has been considered an “adaptation lap,” while the data recorded during the second and third laps have been taken into account for the analysis. Finally, during the third lap two equal events have been simulated both along the Easy and the Hard segment (i.e., four events in total for each subject for each task, Rush and Normal).

**FIGURE 2:**
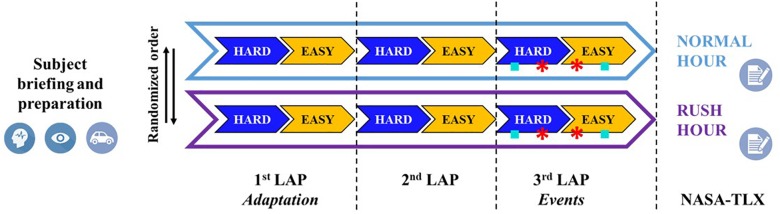
Overview of the experimental protocol, consisting of two main driving tasks different in terms of traffic (normal and rush hour) and performed in a randomized order. Each task consisted of three laps along the circuit in Figure 1: the first lap aimed to allow the driver to take confidence with the circuit, while the second and third lap have been used for analysis. In particular, during the third lap four events have been acted as indicated in Figure 1. Before the experiment the participant received a briefing and was equipped by EEG and Eye Tracking devices, while his car with Video VBOX system. At the end of each task the participant had to fill a questionnaire (NASA-TLX) about the experienced mental workload.

The Figure 2 shows a graphical representation of the experimental protocol.

**FIGURE 3:**
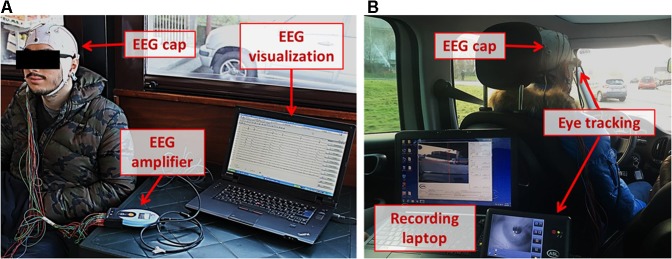
On the left **(A)**, the participant preparation phase. In particular, the EEG signal has been acquired through the EEG amplifier in *holter* modality: the EEG signal and the electrodes impedances were checked on a computer before starting the experiments. On the right **(B)**, a picture representing the experimental setup within the car: in particular, other than the EEG cap, also the Eye Tracking device and its recording laptop are shown. The subject in picture, as all the participants, gave their signed authorization to use the video graphical material for dissemination purposes.

During the whole protocol physiological data, in terms of brain activity through Electroencephalographic (EEG) technique and eye gazes through ET devices, and data about driving behavior, through a professional device mounted on the car (i.e., a VBOX Pro), have been recorded. In addition, subjective measures of perceived Mental Workload have been collected from the subjects after both the tasks through the NASA Task Load Index (NASA-TLX) questionnaire (Hart and Staveland, 1988). It was possible to use Eye Tracker just with half of the subjects’ sample (i.e., 10 subjects) because of device availability, so eye tracker–related data have been analyzed for 10 subjects. The following paragraphs will describe in detail the collection and processing of the aforementioned data, while the Figure 3 shows the subject preparation and the recording setup within the car.

### The Data Collection

#### Electroencephalographic Signal Recording and Processing

The EEG signals have been recorded using the digital monitoring BEmicro system (EBNeuro, Italy). Twelve EEG channels (FPz, AF3, AF4, F3, Fz, F4, P3, P7, Pz, P4, P8, and POz), placed according to the 10–20 International System, were collected with a sampling frequency of 256 Hz, all referenced to both the earlobes, grounded to the Cz site, and with the impedances kept below 20 kΩ. During the experiments the EEG data have been recorded without any signal conditioning, the whole processing chain has been applied offline. In particular, EEG signal has been firstly band-pass filtered with a fourth-order Butterworth filter (high-pass filter cut-off frequency: 1 Hz, low-pass filter cut-off frequency: 30 Hz). The Fpz channel has been used to remove eyes-blink contributions from each channel of the EEG signal by using the REBLINCA algorithm (Di Flumeri et al., 2016). This step is necessary because the eyes-blink contribution could affect the frequency bands correlated to the mental workload, in particular the theta EEG band. This method allows to correct EEG signal without losing data.

For other sources of artifacts (i.e., environmental noise, drivers’ movements, etc.), specific procedures of the EEGLAB toolbox (Delorme and Makeig, 2004) have been employed. Firstly, the EEG signal is segmented into epochs of 2 s (Epoch length), through moving windows shifted of 0.125 s (Shift), thus with an overlap of 0.875 s between two contiguous epochs. This windowing has been chosen with the compromise to have both a high number of observations, in comparison with the number of variables, and to respect the condition of stationarity of the EEG signal (Elul, 1969). In fact, this is a necessary assumption in order to proceed with the spectral analysis of the signal. The EEG epochs with the signal amplitude exceeding ±100 μV (*Threshold criterion*) are marked as “artifact.” Then, each EEG epoch has been interpolated in order to check the slope of the trend within the considered epoch (*Trend estimation*). If such a slope is higher than 10 μV/s, the considered epoch is marked as “artifact.” Finally, the signal sample-to-sample difference (*Sample-to-sample criterion*) has been analyzed: if such a difference, in terms of absolute amplitude, is higher than 25 μV, i.e., an abrupt variation (no-physiological) happened, the EEG epoch is marked as “artifact.” At the end, the EEG epochs marked as “artifact” have been removed from the EEG dataset with the aim to have a clean EEG signal to perform the analyses.

From the clean EEG dataset, the Power Spectral Density (PSD) has been calculated for each EEG channel for each epoch using a Hanning window of the same length of the considered epoch (2 s length, that means 0.5 Hz of frequency resolution). Then, the EEG frequency bands of interest has been defined for each subject by the estimation of the *Individual Alpha Frequency* (IAF) value (Klimesch, 1999). In order to have a precise estimation of the alpha peak and, hence of the IAF, the subjects were been asked to keep the eyes closed for a minute before starting the experimental tasks. Finally, a spectral features matrix (EEG channels × Frequency bins) has been obtained in the frequency bands directly correlated to the mental workload. In particular, only the theta band [IAF – 6 ÷ IAF – 2], over the EEG frontal channels, and the alpha band [IAF – 2 ÷ IAF + 2], over the EEG parietal channels, were considered as variables for the mental workload evaluation (Gevins and Smith, 2003; Aricò et al., 2016b; Borghini et al., 2017a).

At this point the automatic-stop-StepWise Linear Discriminant Analysis (asSWLDA), a specific Machine-Learning algorithm (basically an upgrade version of the well-known StepWise Linear Discriminant Analysis) previously developed (Aricò et al., 2016b), patented (Aricò et al., 2017a) and applied in different applications (Aricò et al., 2016a; Borghini et al., 2017b,c) by the authors has been employed. On the basis of the calibration dataset, the asSWLDA is able to find the most relevant spectral features to discriminate the Mental Workload of the subjects during the different experimental conditions (i.e., EASY = 0 and HARD = 1). Once identified such spectral features, the asSWLDA assigns to each feature specific weights (*w_i train_*), plus a bias (*b_train_*), such that an eventual discriminant function computed on the training dataset [*y_train_(t)*] would take the value *1* in the hardest condition and *0* in the easiest one. This step represents the calibration, or “*Training phase”* of the classifier. Later on, the weights and the bias determined during the training phase are used to calculate the Linear Discriminant function [*y_test_(t)*] over the testing dataset (*Testing phase*), that should be comprised between 0 (if the condition is Easy) and *1* (if the condition is Hard). Finally, a moving average of 8 s (8MA) is applied to the *y_test_(t)* function in order to smooth it out by reducing the variance of the measure: its output is defined as the *EEG-based Workload index (WL*_SCORE_*)*. For the present work, the training data consisted in the *Easy* segment of the 2nd lap during the *Normal* condition and the *Hard* segment of the 2nd lap during the *Rush* condition (they have been hypothesized the two conditions characterized by respectively the lowest and highest mental workload demand), while the testing data consisted of the data of the 3rd lap of both the conditions.

Here below the training asSWLDA discriminant function (Equation 1, where *f_i train_(t)* represents the PSD matrix of the training dataset for the data window of the time sample *t*, and of the *i^th^* feature), the testing one (Equation 2, where *f_i test_(t)* is as *f_i train_(t)* but related to the testing dataset) and the equation of the *EEG-based workload index* computed with a time-resolution of 8 s (*WL_SCORE_*, Equation 3), are reported.

(1) $$y_{\mathrm{train}}(t)=\sum_{i}w_{\mathrm{i train}}\cdot f_{\mathrm{i train}}(t)+b_{\mathrm{train}}$$

(2) $$y_{\mathrm{test}}(t)=\sum_{i}w_{\mathrm{i train}}\cdot f_{\mathrm{i train}}(t)+b_{\mathrm{train}}$$

(3) $${\mathrm{WL}}_{\mathrm{SCORE}}=8\mathrm{MA}(y_{\mathrm{test}}(t))$$

#### Eye-Tracking Data and Its Processing

Eye movements of the participants have been recorded through an ASL Mobile Eye-XG device (EST GmbH, Germany), a system based on lightweight eyeglasses equipped with two digital high-resolution cameras. One camera recorded the scene image and the other the participant’s eye, that is monitored through infrared rays. The data were recorded with a sampling rate of 30 Hz (i.e., 33 ms time resolution), and a spatial resolution of 0.5 ÷ 1°. ASL software was used to analyze the data, obtaining information about the drivers’ fixation points frame by frame (33 ms). A preliminary calibration procedure was carried out for each subject inside the car before starting driving, asking them to fix their gaze on thirty fixed visual points spread across the whole scene, in order to get a good accuracy of the eye-movement recorder. The gazes recorded during the driving task were manually analyzed, in order to group them into three different categories: road infrastructure, traffic vehicles, and external environment. For each subject, each lap (second and third), and each condition (Easy and Hard ROAD, Rush and Normal HOUR) the distribution of eye fixations between the three categories was calculated in terms of percentage of the total.

#### Additional Measures

Each car has been equipped with a Video VBOX Pro (Racelogic Ltd., United Kingdom), a system able to continuously monitor the cinematic parameters of the car, integrated with GPS data and videos coming from up to four high-resolution cameras. The system has been fixed within the car, at the center of the floor of the back seats, in order to put it as close as possible to the car barycenter, while two cameras have been fixed over the top of the car. The system recorded car parameters (e.g., speed, acceleration, position, etc.) with a sampling rate of 10 Hz. For the purpose of the present study, the average speed for each task has been computed. Also, the cameras’ videos have been used to count the number of vehicles encountered by the driver during each task.

Also, at the end of each task (thus only the HOUR condition, i.e., Rush vs. Normal, can be compared) the subjects had to evaluate the experienced workload by filling the NASA-TLX questionnaire (Hart and Staveland, 1988). In particular, the subject had (i) to assess, on a scale from 0 to 100, the impact of six different factors (i.e., Mental demand, Physical demand, Temporal demand, Performance, Effort, Frustration), and (ii) to assess the more impacting factor through 15 comparisons between couple of the previously evaluated factors. The result of this questionnaire is a score from 0 to 100 corresponding to the driver’s mental workload perception.

### Performed Analyses

#### Validation of Experimental Design Assumptions

The first analysis aimed to validate the assumptions in terms of experimental design, that is:

(i) The subjects drove during two conditions different in terms of traffic, i.e., Rush and Normal hour;
(ii) The circuit was constituted by two segments different in terms of road complexity, thus in terms of difficulty, i.e., Hard and Easy.

In order to validate the first assumption, the number of vehicles encountered by the experimental subjects and the average driving speed during the two conditions have been computed and statistically compared. It is expected that the number of vehicles is significantly higher and the average speed significantly lower during rush hours (Bucchi et al., 2012).

The second assumption has been validated by investigating the percentage of fixations over the external environment, since such indicator has been proven to be inversely correlated with mental workload: the more the experienced workload is, the less the number of fixations over the external environment is, since the driver gaze will mostly focus on infrastructure and vehicles (Costa et al., 2014; de Winter et al., 2014; Lantieri et al., 2015). Also, we verified the difference in terms of mental workload from a neurophysiological point of view: we computed the ratio between Theta rhythms over frontal sites (“*ThetaF”*) and Alpha rhythms over parietal sites (“*AlphaP”*), since it is considered a well-established metric of mental workload (Borghini et al., 2014). In particular, The *ThetaF/AlphaP* has been proven to increase if the mental workload experienced by the user is increasing as well (Gevins and Smith, 2003; Holm et al., 2009; Borghini et al., 2015). The metric has been computed as the ratio between the averaged PSD values in theta band over the frontal electrodes (AF3, AF4, F3, Fz, F4) and the averaged PSD values in alpha band over the parietal electrodes (P3, P7, Pz, P4, P8, POz). Both the analysis have been performed comparing the two conditions employed to train the classifier (please see Electroencephalographic Signal Recording and Processing), i.e., the *Easy* segment of the 2nd lap during the *Normal* condition and the *Hard* segment of the 2nd lap during the *Rush* condition, assumed as the two conditions characterized by respectively the lowest and highest mental workload demand.

All the statistical comparisons have been performed through two-sided Wilcoxon signed rank tests. In fact, data come from multiple observations on the same subjects, but it is not possible to assume or robustly assess (the number of observations is always equal or less than 16) that the observations distribution is Gaussian, therefore paired non-parametric tests have been used (Siegel, 1956).

#### Classification Performance

Firstly, a synthetic analysis of the brain features selected by the algorithm has been performed in order to evaluate any eventual recurrence of a specific feature. The initial features domain for each subject consisted in a matrix of 187 features (11 EEG channels ^∗^ 17 bins of frequency – from IAF-6 Hz to IAF+2 Hz with a resolution of 0.5 Hz –). Actually, only 99 of these features can be selected by the algorithm because of the Regions of Interest defined *a priori*: 45 features related to frontal Theta and 54 related to parietal Alpha.

Then, in order to investigate the algorithm (i.e., the asSWLDA) classification accuracy, the analysis of the Area Under Curve (AUC) of the Receiver Operator Characteristic (ROC) curve of the classifier has been performed (Bamber, 1975). In particular, AUC represents a widely used methodology to test the performance of a binary classifier: the classification performance can be considered good with an AUC higher than at least 0.7 (Fawcett, 2006). In this case there are actually two classes in terms of mental workload, i.e., Easy and Hard, related to the two different difficulty levels characterizing the circuit. As previously described, for each subject the training dataset consisted in the Easy segment of the 2nd lap during the Normal condition and the Hard segment of the 2nd lap during the Rush condition (they have been hypothesized the two conditions characterized by respectively the lowest and highest cognitive demand), while the testing dataset consisted of the data of the 3rd lap of both the conditions (*Real data*). Therefore, the classifier has been tested shuffling the testing dataset related labels (*Random*), in order to verify that classifier performance on measured data (*Real data*) was significantly higher than that one obtained on random data (*Random*), independently from the traffic intensity (i.e., both in Rush and Normal hour conditions). In both the cases (Real and Random), the time resolution of WL_scores_ is equal to 8 s, obtained as the best compromise between a high time resolution and good classification performance. Three two-sided Wilcoxon signed rank tests have been performed between *Real* and *Random data*, one for each HOUR condition (i.e., comparison Real vs. Random in *Normal* and *Rush* hour) and one comparing the *Normal* and *Rush* conditions only in terms of real data. The results of these multiple comparisons have been validated by applying the False Discovery Rate (FDR) correction (Benjamini and Hochberg, 1995).

#### Workload Assessment

Once demonstrated the reliability of the classification algorithm to obtain the EEG-based index of mental workload in the specific driving scenarios, the workload scores (*WL score*) have been used to evaluate the impact of different factors, that is the road complexity and the traffic as well as specific events along the driving experience. Depending on the analysis, the EEG-based WL scores have been analyzed in relation to ET and subjective data.

##### Evaluation of traffic and road complexity impact

The WL indexes obtained with a time resolution of 8 s from the testing dataset (i.e., the third lap) were averaged for each subject and for each condition (i.e., HOUR and ROAD). A Friedman test, the non-parametric version of the repeated measures ANOVA (Analysis of Variance), has been performed in order to investigate any possible effect due to traffic and road complexity on the workload perceived by the subject. Furthermore, since *post hoc* tests specifically designed for Friedman test do not exist but both the factors have been measured on the same subjects, two Wilcoxon signed rank tests have been performed in order to investigate potential within effects among the two factors, i.e., HOUR and ROAD.

Also, the results in terms of workload indexes have been compared with those obtained from ET in order to evaluate the different sensitivity to the phenomenon (i.e., mental workload variations) of the two technologies. In terms of ET measures, it has been investigated the percentage of fixations on the road infrastructure and vehicles, since such indicator has been proven to be directly correlated with mental workload while driving: the more the experienced workload is, the more the number of fixations over the road will be, since the driver gaze will mostly focus on infrastructure and vehicles (Costa et al., 2014; de Winter et al., 2014). Multiple two-sided Wilcoxon signed rank tests have been performed in order to reveal any difference with respect to the two investigated factors.

Furthermore, a two-sided Wilcoxon signed rank test has been performed on the NASA-TLX measures. Please note that for the continuity of the experiment the questionnaires were filled by the subjects only after the tasks end, therefore only the comparison between Normal and Rush hour has been possible (please refer to Section “Additional Measures”).

##### Evaluation of single events impact

On the basis of the average duration of the events among the subjects during the driving experience, and to homogenize the measures with respect of this parameter (i.e., event duration), a fixed window of 20 s for the car event (from the first fixation of the car to its overtaking) and of 10 s for the pedestrian event (from the first fixation of the pedestrian to the acceleration after its road crossing) has been defined, independently from the traffic and the road complexity. Remembering that the events were acted only during the third lap of each task repetition, similar windows corresponding to the same circuit position were defined during the second lap in order to compare the event’s happening vs. no-happening. The WL indexes were averaged for each subject, for each condition (i.e., HOUR and ROAD) and for each event. Multiple two-sided Wilcoxon signed rank tests have been performed in order to reveal any difference (i) with respect to the events’ happening, and (ii) among the events types.

## Results

The following results are referred to a sample of 16 subjects (8 with Eye Tracking), since one subject has been discarded because of technical issues on the EEG data, while three subjects have been discarded because of no objective difference in terms of encountered vehicles (measured through the VBOX cameras) between the two tasks, i.e., during Rush and Normal hours.

### Experimental Design Validation

**FIGURE 4:**
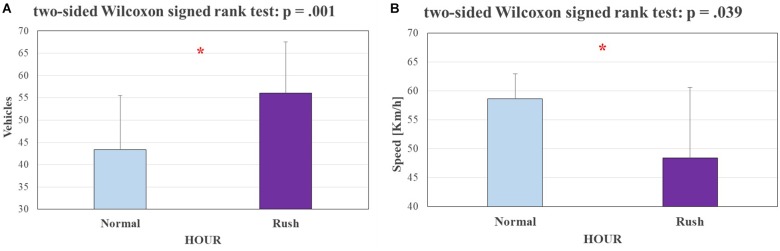
On the left **(A)**, a bar graph representing the mean and the standard deviation of vehicles encountered by the participants during the experiments. The Wilcoxon test showed a significantly higher (*p* = 0.001) number of vehicles during rush hour. On the right **(B)**, a bar graph representing the mean and the standard deviation of participants driving speed during the experiments. The Wilcoxon test showed a significantly lower (*p* = 0.039) speed during rush hour. The statistical tests showing a significant effect.

Figure 4 shows the results of the comparisons between (a) the number of vehicles encountered by the experimental subjects and (b) the average driving speed during the two different traffic conditions, i.e., during *Normal* and *Rush* hours. The performed statistical analysis revealed a significant increasing (*p* = 0.001) of vehicles encountered by the experimental subjects and a significant decreasing (*p* = 0.039) of driving average speed from *Normal* to *Rush* hours, validating the experimental hypothesis about the two different conditions of traffic made *a priori* on the basis of the General Plan of Urban Traffic of Bologna (see The Experimental Protocol).

**FIGURE 5:**
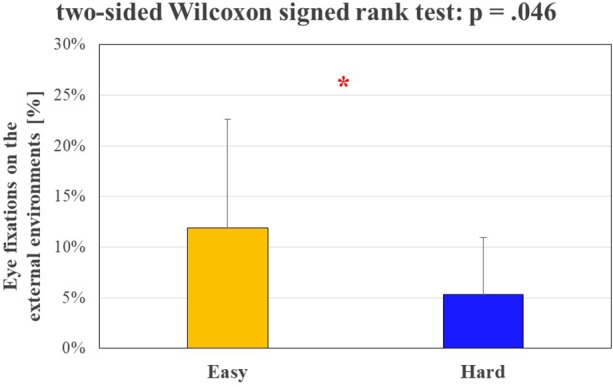
The bar graph represents the mean and the standard deviation of the percentage of drivers’ eye fixations along the two different segments of the circuit. The Wilcoxon test showed a significant reduction (*p* = 0.046) of such percentage during the circuit segment characterized by hard complexity. The statistical tests showing a significant effect.

Figure 5 shows the results in terms of percentage of fixations over the external environment between the *Easy* and *Hard* segments of the circuit, since such indicator has been proven to be inversely correlated with mental workload. The performed statistical analysis revealed a significant decreasing (*p* = 0.046) of driver gazes over the external environment, validating the experimental hypothesis about the two different conditions of difficulty made *a priori* on the basis of scientific literature (see The Experimental Protocol).

**FIGURE 6:**
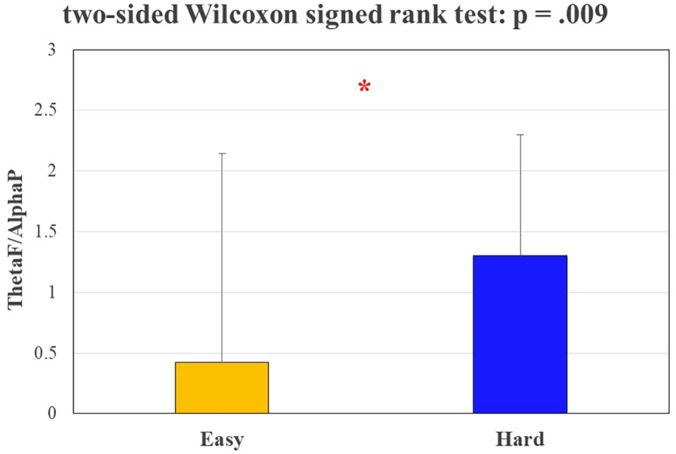
The bar graph represents the mean and the standard deviation of the EEG-based ThetaF/AlphaP indicator along the two different segments of the circuit. The Wilcoxon test showed a significant increasing (*p* = 0.009) of such indicator during the circuit segment characterized by hard complexity. The statistical tests showing a significant effect.

Figure 6 shows the results in terms of *ThetaF/AlphaP* value between the *Easy* and *Hard* segments of the circuit, since such ratio has been proven to be a physiological indicator directly correlated to mental workload. The performed statistical analysis revealed a significant increasing (*p* = 0.009) of the proposed index, validating the assumption about the different cognitive demand related to the two conditions, made *a priori* on the basis of scientific literature (see The experimental Protocol).

### Classification Performance

**FIGURE 7:**
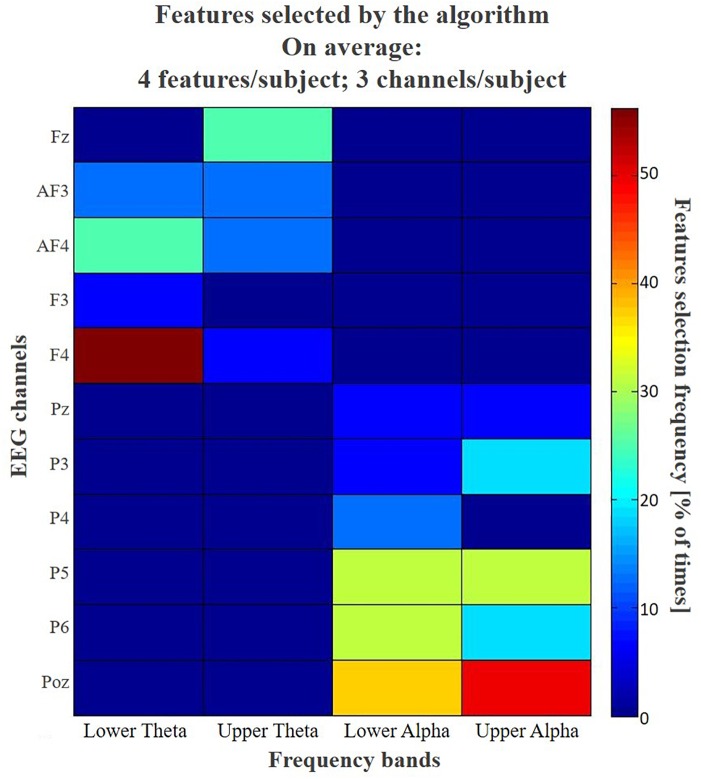
The colormap summarizes the frequency of the selection of each feature for the whole subjects’ sample. The initial features domain for each subject consisted of a matrix of 187 features (11 EEG channels ^∗^ 17 bins of frequency – from IAF – 6 Hz to IAF+2 Hz with a resolution of 0.5 Hz –). Actually, only 99 of these features can be selected by the algorithm, because of the Regions of Interest defined *a priori*: 45 features related to frontal Theta and 54 related to parietal Alpha. For a synthetic and effective representation, the frequency bins, actually equal to 17 because included between IAF-6 Hz and IAF+2 Hz with a resolution of 0.5 Hz, have been grouped into four areas of interest: Lower Theta [IAF – 6 ÷ IAF – 4], Upper Theta [IAF – 4 ÷ IAF – 2], Lower Alpha [IAF – 2 ÷ IAF] and Upper Alpha [IAF ÷ IAF + 2]. The results show that Lower Theta over F4 and Upper Alpha over POz have been used for more than the 50% of subjects.

Figure 7 shows the distribution of the features, and the relative frequency of selection, chosen by the asSWLDA during the training phase. The analysis of features selected by the algorithm revealed that the asSWLDA selected on average 4 features per subject, coming from 3 of the 11 channels available. The frequency bins, actually equal to 17 because included between IAF-6 Hz and IAF+2 Hz with a resolution of 0.5 Hz, have been grouped into four areas of interest: Lower Theta [IAF – 6 ÷ IAF – 4], Upper Theta [IAF – 4 ÷ IAF – 2], Lower Alpha [IAF – 2 ÷ IAF] and Upper Alpha [IAF ÷ IAF + 2]. The results show that Lower Theta over F4 and Upper Alpha over POz have been used for more than the 50% of subjects.

**FIGURE 8:**
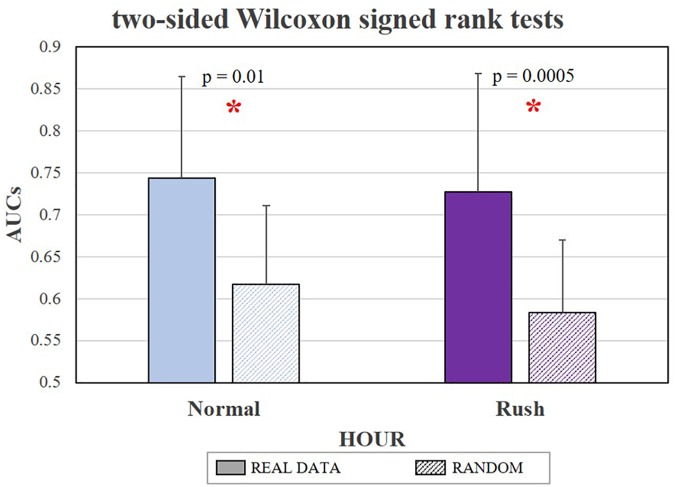
The bar graph represents the mean and the standard deviation of AUC values obtained in discriminating the Easy and Hard circuit segments. Both in Normal and Rush hour conditions, the classification performance obtained by training the classifier with real data (solid color) have been significantly higher (respectively *p* = 0.01 and *p* = 0.0005) than by using random data (lines pattern), achieving mean AUC values of respectively 0.74 and 0.73. The statistical tests showing a significant effect.

The AUC analysis (Figure 8) revealed that, by using such approach, it has been possible to achieve mean AUC values of 0.744 ± 0.13 for the *Normal* hour and of 0.727 ± 0.06 for the *Rush* hour. In particular, the two Wilcoxon tests demonstrated that the classifier performance on the Real data was significantly higher than on Random data in both the conditions (respectively *p* = 0.01 and *p* = 0.0005). Also, there were no significant differences (*p* = 0.64) in terms of AUC values on Real data between *Normal* and *Rush* hours, in other words the classification performance was not dependent on the traffic condition. Because of the three repeated tests, the False Discovery Rate correction has been performed: with respect to the *p*-values obtained and ordered (0.0005, 0.01, and 0.64), the three corrected *q*-values are respectively 0.0015, 0.015, and 0.64, thus the first two results are still significant.

### Workload Assessment

#### Evaluation of Traffic and Road Complexity Impact

**FIGURE 9:**
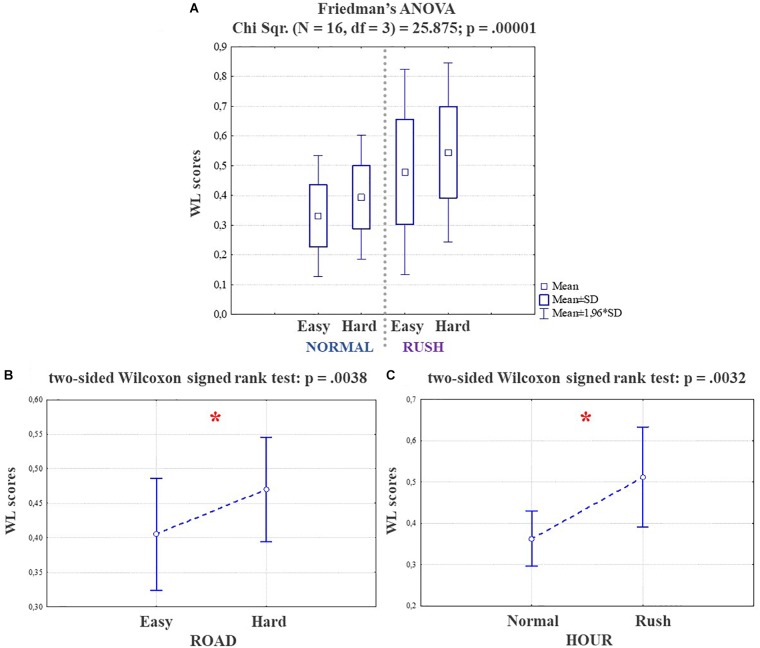
At the top **(A)**, the Friedman test highlighting a significant main effect (*p* = 0.00001), in terms of mental workload increasing among the different factors. At the bottom, on the left **(B)** the Wilcoxon test on the factor ROAD and on the right **(C)** the same test on the factor HOUR, showing how both the factors produced a significant mental workload increasing (respectively *p* = 0.004 and *p* = 0.003). The statistical tests showing a significant effect.

Figure 9 shows the results of the non-parametrical statistical analysis in terms of effects of the two investigated factors, i.e., the traffic (HOUR) and the road complexity (ROAD), on the mental workload experienced by the drivers. In particular, the Friedman test at the top of Figure 9A highlights a significant main effect (*p* = 0.00001) among the different factors: the mental workload significantly increased because of the higher road complexity (i.e., from Easy to Hard), and even more because of the higher traffic intensity (i.e., from Normal to Rush hours). The Wilcoxon tests performed in order to investigate any *within* effect showed two significant main effects in term of workload increasing if both complexity [bottom left (Figure 9B), ROAD, *p* = 0.0038] and traffic [bottom right (Figure 9C), HOUR, *p* = 0.0032] increase.

**FIGURE 10:**
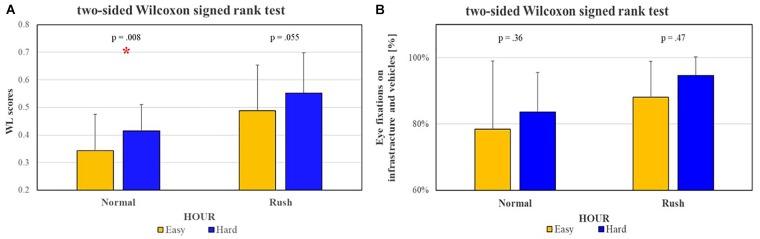
The Wilcoxon tests performed to investigate eventual sensitivity differences between Eye-Tracking [left **(A)**] and EEG [right **(B)**] measures, considered on the same subjects, in relation to ROAD complexity showed that EEG-based measures have been able to significantly discriminate (*p* = 0.008) the two conditions at least during Normal hour, while the ET-based ones have not been able to show any significant difference both during Normal and Rush hours. The statistical tests showing a significant effect.

**FIGURE 11:**
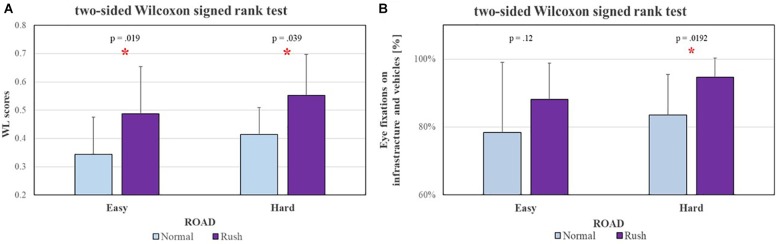
The Wilcoxon tests performed to investigate eventual sensitivity differences between Eye-Tracking [left **(A)**] and EEG [right, **(B)**] measures, considered on the same subjects, in relation to traffic intensity (i.e., HOUR) showed that, while the EEG-based measures have been able to significantly discriminate the two conditions both along Easy (*p* = 0.019) and Hard (*p* = 0.04) segments, the ET-based ones have been able to significantly discriminate Normal and Rush hours only along the Hard segment (*p* = 0.02). The statistical tests showing a significant effect.

Figures 10, 11 show the results of the Wilcoxon tests comparing the sensitivity of ET measures with respect to EEG-based ones. For these analyses the EEG-based WL scores of only the subjects wearing also the Eye Tracker (eight of sixteen) have been considered, in order to make the results comparable (i.e., both the measures have been collected during same experience). In particular:

• Figure 10: in terms of ROAD complexity, while the EEG-based measures have been able to significantly discriminate (*p* = 0.008) the two conditions at least during Normal hour, the ET-based ones have not been able to show any significant difference both during Normal and Rush hours;
• Figure 11: in terms of traffic HOUR, while the EEG-based measures have been able to significantly discriminate the two conditions both along Easy (*p* = 0.019) and Hard (*p* = 0.039) segments, the ET-based ones have been able to significantly discriminate Normal and Rush hours only along the Hard segment (*p* = 0.0192).

**FIGURE 12:**
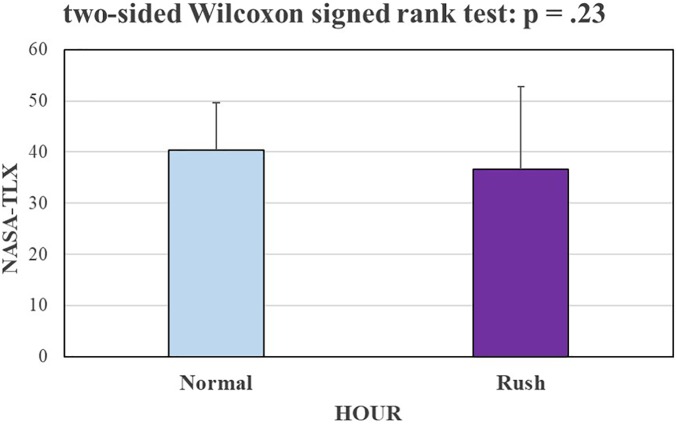
The bar graph represents the mean and the standard deviation of NASA-TLX scores, i.e., the subjective assessment of the mental workload experienced by the participants of the circuit. The Wilcoxon test does not reveal any significant difference in terms of workload subjectively assessed by the subjects between the Normal and Rush hour conditions.

Finally, Figure 12 shows the results in terms of NASA-TLX scores, revealing that there is not any significant difference in terms of workload subjectively assessed between the *Normal* and *Rush* hour conditions.

#### Evaluation of Single Events Impact

**FIGURE 13:**
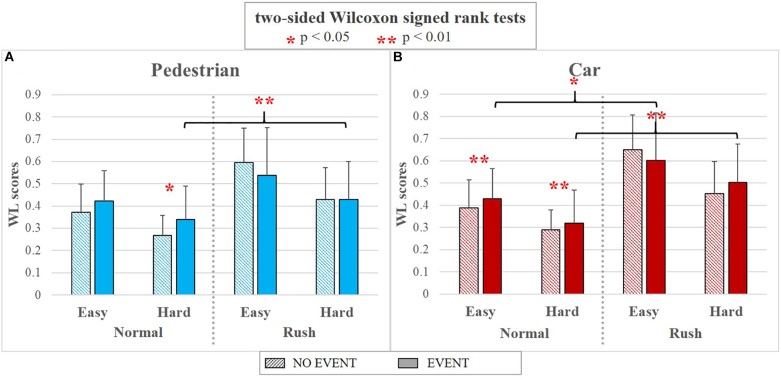
The bar graphs show the mean values and the standard deviation of the EEG-based WL scores related to the different events along the various experimental conditions. In particular, the results are divided per events category, i.e., Pedestrian on the left **(A)** and Car on the right **(B)**. In both the cases, the condition EVENT (solid color) is referred to the event actually happened during the 3rd lap, the condition NO EVENT (lines pattern) is referred to the same circuit portion during the 2nd lap when no events were acted. The Wilcoxon tests revealed a significant workload increasing (one red asterisk stands for *p* < 0.05; two red asterisks stand for *p* < 0.01) related to both the investigated events, that is the car and the pedestrian crossing the road, especially in normal hours independently from the road complexity. Instead, no significant workload increasing were associated to the event during rush hours, despite a significantly higher workload in comparison with the same events during normal hours.

Figure 13 shows the results in terms of EEG-based WL scores about how the presence of a specific event impacts the mental workload of the driver, with respect to the different experimental conditions. In terms of external events (the condition *EVENT* is referred to the event actually happened during the 3rd lap, the condition *NO EVENT* is referred to the same circuit portion during the 2nd lap when no events were acted), the pedestrian crossing the road induced a significantly higher workload only during the *Normal* hour along the *Hard* circuit segment (Wilcoxon test’s *p* = 0.037), while the car induced a significantly higher workload along both the *Easy* and *Hard* circuit segments but only during *Normal* hour (respectively Wilcoxon test’s *p* = 0.007 and *p* = 0.008).

Considering only the condition “EVENT,” despite a decreasing trend from *Easy* to *Hard* segments, no significant differences (*p* > 0.05) have been found for each event during the same traffic condition (HOUR). However, if considering the same difficulty level (ROAD), all the events induced a significant workload increasing during *Rush* hours, except the pedestrian along the Easy segment (Pedestrian *Hard*: *p* = 0.009; Car *Easy*: *p* = 0.023; Car *Hard*: *p* = 0.002).

## Discussion

Since the impact of drivers’ errors in terms of human lives and costs is very high and the next future previsions are even worse (World Health Organization, 2015), the relationship between human errors and driving performance impairment due to a high mental workload has been deeply investigated in the automotive domain. Recent technological advancements as well as the growth of disciplines such as Neuroscience and Neuroergonomics now allow to record human neurophysiological signals, such as in this study brain activity through Electroencephalographic technique, in a robust way also outside the laboratory, and to obtain from them objective neurometrics of human mental states (i.e., workload) (Aricò et al., 2017c, 2018). The present work aimed to validate a machine-learning approach, i.e., the asSWLDA (Aricò et al., 2016a), for the objective assessment of human mental workload while driving in real settings, as well as its integration with traditional tools (e.g., questionnaires, car parameters, eye tracking) in order to evaluate the impact of different factors (road complexity, traffic intensity, external events), thus suggesting new innovative tools for enhancing research in road safety. In order to achieve these objectives, 20 young subjects have been involved in a real driving task along urban roads, performed under different traffic conditions (rush hour and not), driving through different road types (main and secondary streets) and facing to external events.

Firstly, the experiments have been designed making two *a priori* assumptions:

(1) the experiments have been conducted in two different conditions of traffic intensity, depending on the hours (i.e., normal and rush hour) of the day; the experimental design initially referred to the General Plan of Urban Traffic of Bologna;
(2) the circuit consisted of two segments of different difficulty, i.e., Easy and Hard, because of the related road complexity (Harms, 1991; Verwey, 2000; Paxion et al., 2014).

The statistical analysis performed on the average speed of the experimental subjects and the number of vehicles encountered during the experiments (Figure 4) validated the Assumption 1: in fact, the subjects encountered a significantly higher (*p* = 0.001) number of vehicles and they drove at a significantly lower (*p* = 0.039) speed during the rush hours, as expected from scientific literature (Bucchi et al., 2012). Statistical analysis of driver’s eye fixations over the external environment (Figure 5) and physiological brain patterns (Figure 6) validated the Assumption 2: in fact the drivers’ gazes over the external environment (such index inversely correlates with mental workload; de Winter et al., 2014; Lantieri et al., 2015) have been significantly lower (*p* = 0.046) along the circuit segment that was hypothesized as *Hard*, while the ratio between frontal theta and parietal alpha rhythms significantly increased (*p* = 0.009). These results confirmed the properness of the experimental design. Nevertheless, the analysis of encountered vehicles, determined through videos from the VBOX videos, led to discard three subjects because of no differences between rush and normal hours (see Results). Therefore, this validation approach should be taken into account for future works in real driving conditions, where external conditions and events are less controllable, even unpredictable, if compared with laboratory experiments.

Once validated the experiment in terms of differences between the road and traffic conditions, the EEG-based Workload measures have been validated. In particular, the analysis of AUC related to the asSWLDA-based classifier demonstrated that the adopted approach achieves considerable performance, i.e., AUCs > 0.7 (Fawcett, 2006). More in detail, the AUC analysis (Figure 8) revealed that it has been possible to achieve mean AUC values of 0.74 for the Normal hour and of 0.73 for the Rush hour, significantly higher than a random classification in both the conditions (respectively *p* = 0.01 and *p* = 0.0005). Also, there were no significant differences (*p* = 0.64) in terms of AUC values on Real data between Normal and Rush hours. All the previous results have been also confirmed by the correction for multiple comparisons, in this case the False Discovery Rate. It is also true that, within the machine-learning theory, AUCs greater than 0.7 are considered remarkable if compared with a random distribution that is assumed to produce AUCs equal to 0.5. In the present study, the performance of the classifier on randomized data achieved AUCs values of about 0.6. A possible explanation could be that the random value would be closer and closer to 0.5 only if the number of repetitions tends to infinite, however, this result undoubtedly encourages research about improving the proposed method. Of course, classification performance of about 0.75 are anyway remarkable, in particular because of the novelty of such application (the EEG-based Workload index is provided with a time resolution equal to 8 s) and the real settings, where mental states assessment is more prone to misclassification: in fact, it is plausible to assume that outside the high controlled laboratory settings, the user experiences more complex mental states that consist of multiple different components having the potential to influence neurophysiological signals used to infer a specific state.

The analysis of the patterns of features selected by the algorithm during its training phase (Figure 7) provided interesting insights about its usability: in fact, the asSWLDA selected on average 4 discriminant features for each subject, and even more interesting, by involving 3 of the 11 available channels. It means that, once calibrated the system on a specific user, it would be able to work online during the driving experience involving only three EEG channels, in other words reducing significantly its invasiveness and increasing wearability, two critical aspects for applications outside the laboratory.

At this point, the asSWLDA output, in terms of EEG-based Workload index, has been used to evaluate the effects of road complexity, traffic intensity and external events on drivers’ workload (Figure 9).

The Friedman ANOVA test (please see Figure) shows the results in terms of effects of the two investigated factors, i.e., the traffic (HOUR) and the road complexity (ROAD), on the mental workload experienced by the drivers: both the traffic and road complexity contributed to significantly increase (main effect: *p* = 0.00001; Wilcoxon tests respectively: HOUR, *p* = 0.0032; ROAD: *p* = 0.0038) the mental workload. In other words, the drivers’ workload increased if traffic increased as well independently from the road complexity. At the same time, the drivers’ workload increased while driving along more complex roads independently from the traffic intensity. These results have to be considered with respect to the experimental task: actually, the *Hard* segment was a three-lanes main street, that with respect to a one-lane main street (*Easy* segment) implies several additional decisions and actions, such as eventual car overtaking as well as looking at rear-view mirrors because of possible cars coming on lateral lanes. Of course, these actions increase with traffic increasing, because of the higher number of vehicles along the circuit (as demonstrated by Video analysis, please see Figure 4). Apparently, the Easy segment should not suffer traffic increasing, since being a one-lane segment the overtaking are very limited and drivers have not to frequently check rear-view mirrors since they cannot change lane. Nevertheless, because of the higher number of vehicles along the circuit during rush hours, the drivers had to continuously monitor eventual preceding cars, adapting safety distance and speed (in fact average speed during rush hour has been lower and drivers’ gazes on infrastructure and vehicles higher also along Easy segment). These actions also induced a no-negligible workload increasing, giving a possible justification of the high accident rate along rural roads (Shankar et al., 1995), that are generally considered “*Easy to drive*” if compared with urban main roads (Harms, 1991; Paxion et al., 2014), thus mismatching the driver’s expectations.

Very interestingly but not surprisingly, the neurophysiological measures showed a significantly higher sensitivity with respect to the ET ones (Figures 10, 11) in discriminating the different impact of road complexity and traffic intensity on mental workload. It is important to consider that ET measures were available only for a reduced group of the experimental sample (8 of 16 subjects), therefore it could have affected the performance of such measures in discriminating the mental workload related to different factors. However, the paired statistical analysis highlighted that on the same subjects, EEG-based measures were more sensitive to workload fluctuations. Their high sensitivity has been pointed out also with respect to subjective measures (i.e., NASA-TLX questionnaires, Figure 12), that on the contrary were not able to discriminate (*p* = 0.23) normal from rush hours.

Finally, EEG-based workload measures revealed a significant workload increasing (*p* < 0.05) related to both the investigated events, that is the car and the pedestrian crossing the road, especially in normal hours independently from the road complexity. Instead, no significant workload increasing were associated to the event during rush hours, despite a significantly higher workload in comparison with the same events during normal hours (Figure 13). Although for this analysis neurophysiological measures are not integrated with additional ones (it was impossible to collect subjective data related to specific events, while from the ET point of view it was possible to assess only if the event was been perceived or not), it is possible to deduce that external events could lead to eventually risky situations especially with low traffic (normal hours). In fact, although a lower absolute workload if compared with high traffic condition, they are characterized by an immediate cognitive demand increase, that could become dangerous if not expected by the driver.

Nevertheless, the main limit that affects the present study is the algorithm calibration with data coming from the task itself and recorded in very similar conditions. From one side, it could be argued that in everyday life context such a calibration would be unfeasible; from the other side it could be argued that the proposed algorithm is not classifying the targeted mental state, i.e., mental workload, but only two conditions that are very similar. Regarding the calibration, actually it is one of the main still open issues in transferring machine learning approaches from research to applied field: several solutions have been explored, such as cross-task calibration or employment of unsupervised algorithms, but the problem is still open and needs further investigation (Aricò et al., 2018). However, the present work did not aim at addressing such issue, but at investigating the possibility of applying a machine-learning algorithm for the mental workload evaluation, already validated in other domains, also in automotive applications. The highly challenging conditions of a “real driving experiment” with twenty subjects, jointly with the employment of high-quality instrumentation, already make the present work very innovative and of interest. Secondly, it is true that the algorithm has been calibrated on two conditions and employed in classifying two similar conditions, but it is also important to consider that calibration data for each subject came from two different repetitions (please refer to Section “Electroencephalographic Signal Recording and Processing” for more information): in fact data recorded during the *Easy* segment of *Normal* hour (2nd lap) have been used as EASY CLASS, while data recorded during the *Hard* segment of *Rush* hour (2nd lap) have been used as HARD CLASS. Even if assuming that *Easy* segment of *Normal* hour and *Hard* segment of *Rush* hour of 2nd and 3rd lap were intrinsically similar, no data from *Hard* segment of *Normal* hour and *Easy* segment of *Rush* hour have been used to train the classifier, therefore their coherent classification (e.g., *Hard* segment of *Normal* hour is not easier than the *Easy* segment during the same hour) is a mere and appreciable result of the proposed algorithm. Undoubtedly, mental workload is a Human Factor concept hard to define and even worse to measure (Moray, 2013), and confounds arising from different mental states are probably present, however, the results of the present study are already remarkable, especially if considering previous results obtained by the employment of the same algorithm in different applications (Aricò et al., 2016a,b, Borghini et al., 2017b,c).

It is important to remark how it is possible to achieve this kind of results only thanks to the proposed methodology: in fact, subjective measures cannot be gathered with high time resolution and without interfering with the main task, briefing and debriefing sessions can be performed only before and after the experience, while eye-tracker as well as other neurophysiological metrics (for example the *ThetaF*/*AlphaP* showed in Figure 6) are able to provide only an overall evaluation about a “long” condition. On the contrary, the proposed methodology is able to overcome these limitations, providing workload assessment with high time resolution (i.e., in this case 8 s) and thus allowing to evaluate also specific events.

In conclusion, the obtained results appear very interesting in terms of understanding driver’s behaviors and its relationship with road environment, highlighting the added value of neurophysiological measures in providing insights about human mind that are not obtainable, or at least difficult to obtain, with traditional approaches. Certainly, further analyses are necessary in order to validate this multimodal approach with a larger sample of subjects, exploring the impact of other factors, such as different events, road signage and so on, and involving additional tools typical of road safety research, as well as exploring the possibility of calibrating the proposed algorithm without any task-related data.

## Conclusion

The present study, through a real driving experiment, aimed to validate a methodology able to infer driver’s mental workload on the basis of his/her brain activity through Electroencephalographic technique. Once validated, such methodology has been successfully employed to evaluate the impact of different factors, specifically the road complexity, the traffic intensity (depending on the hour of the day), and two specific events (a pedestrian crossing the road and a car entering in the traffic flow), on the drivers’ experienced mental workload. The analyses have been supported by information coming from subjective measures, drivers’ eye movements tracking and car parameters. The results demonstrated (i) the reliability and effectiveness of the proposed methodology based on human EEG signals to objectively measure driver’s mental workload with respect to different road factors, and (ii) the added value of neurophysiological measures in providing insights about human mind while dealing with tasks that are difficult or even impossible to obtain by using traditional approaches. In conclusion, other than the specific obtained results, the present work breaks new ground for the integration of these new methodologies, i.e., neurophysiological measures, with traditional approaches in order to enhance and extend research on drivers’ behaviors and road safety.

## Ethics Statement

This study was carried out in accordance with the recommendations of the Good Clinical Practice (International Council for Harmonisation of Technical Requirements for Pharmaceuticals for Human Use) with written informed consent from all subjects. All subjects gave written informed consent in accordance with the Declaration of Helsinki. The protocol was approved by the ‘University of Bologna.’

## Author Contributions

GDF is the main author of the paper. Also, he was actively involved within the experiments as well as in the EEG data analysis and interpretation. PA, GB, and NS supported the experimental design, the data recording, the EEG data analysis, and the manuscript writing. PL and SP supported the experimental design and the results interpretation, providing their contribute in particular about the Human Factor concepts. VV, CL, AB, and AS were in charge of experiments planning, they contributed actively to the experiments execution, they analyzed Eye Tracking and subjective measures analysis, and supported the results interpretation. FB coordinated the research group, from the experimental design to the manuscript editing.

## Conflict of Interest Statement

The authors declare that the research was conducted in the absence of any commercial or financial relationships that could be construed as a potential conflict of interest.
